# The "*Statinth*" wonder of the world: a panacea for all illnesses or a bubble about to burst

**DOI:** 10.1186/1477-5751-4-3

**Published:** 2005-03-23

**Authors:** Nusrat Shafiq, Samir Malhotra, Promila Pandhi, Anil Grover

**Affiliations:** 1 Department of Pharmacology, Post Graduate Institute of Medical Education and Research, Chandigarh, 160012, India; 2 Department of Cardiology, Post Graduate Institute of Medical Education and Research, Chandigarh, 160012, India

## Abstract

After the introduction of statins in the market as effective lipid lowering agents, they were shown to have effects other than lipid lowering. These actions were collectively referred to as 'pleiotropic actions of statins.' Pleiotropism of statins formed the basis for evaluating statins for several indications other than lipid lowering. Evidence both in favour and against is available for several of these indications. The current review attempts to critically summarise the available data for each of these indications.

## 

Recently while browsing through the internet, we came across a webpage [1] that reads as follows: "Statin drugs should probably be in the water, like fluoride. These cholesterol fighting wonders have been proven to prevent heart attacks..... with only rare side effects............... The hitch is that statins cost more than fluoride. A lot more. The drug industry's statin sales surpassed US $15 billion last year. The cholesterol fighting power of products like Pfizer's Lipitor and Merck's Zocor have won them the title 'Superstatins' and made them supersellers. Lipitor brought in US $9.2 billion in 2003 sales for Pfizer, making it the biggest prescription drug in the world."

In 2001, we reviewed the statin literature for Medscape and were able to enlist about seven indications[2], the major one being dyslipidemia with associated coronary disease (CAD). The 1993 National Cholesterol Education Programme (NCEP) guidelines [3] were cautiously optimistic about the future of statins but subsequent publication of 3 landmark trials [4-6], greatly tilted the balance in their favour and since then they haven't looked back: a large number of trials and guidelines added new intensity to cholesterol lowering with the low density lipoprotein cholesterol (LDL-C) targets going for a free fall (<70 mg/dl in some situations) [7-12]. Although this approach of more intense lipid lowering has met with considerable criticism, this is not the topic of this review. We intend to discuss the other novel, upcoming uses of statins.

In contrast to the post-hoc analysis of the Scandinavian Simvastatin Survival Study (4S) [4] in which the benefit provided was related to the magnitude of change in the LDL-C levels, some other studies have shown benefits that could not be accounted for by reduction in LDL-C alone [13-16]. A large number of studies showing pleiotropism of statins followed and diverse mechanisms were then proposed to explain this pleiotropism including anti-inflammatory, immunomodulating, and effects on apoptosis [17-22], making them potentially suitable candidates for the treatment of a wide variety of pathological conditions in many of which they are already being investigated.

This article attempts to summarize the available evidence for the proposed (other than lipid lowering) indications of statins.

### Arrhythmias

Several actions of lipid lowering therapy like reduction in myocardial ischemia, improvement of autonomic function, changes in protein channel function and inhibition of cardiac remodelling make them prospective agents for the treatment of arrhythmias[22,23]. Chronically administered pravastatin was shown to reduce the incidence of ischemia-induced ventricular tachyarrhythmias in experimental models [24,25]. Early use of pravastatin in patients with acute myocardial infarction (MI) reduced the incidence of in-hospital ventricular arrhythmias irrespective of the lipid levels [26]. The Anti-arrhythmia Versus Implantable Defibrillators (AVID) Study showed that lipid lowering therapy decreased the recurrence rate of ventricular arrhythmias in patients implanted cardioverter-defibrillator [27].

Statins have also been shown to have a role in the treatment of atrial arrhythmias. Inflammatory changes have been shown in atrial biopsy specimens of patients with lone atrial fibrillation (AF) [28]. Furthermore, serum levels of C-reactive protein (CRP), a sensitive marker of systemic inflammation, were increased in patients with AF. Not only that, CRP levels were higher in patients with persistent rather than paroxysmal AF, and persistent AF is less likely to spontaneously revert to sinus rhythm [29,30]. These studies suggested that inflammation may induce, provoke and promote the persistence of AF. Statins may be potent anti-inflammatory agents [31] and have also been shown to reduce CRP levels [32].

Not surprisingly, statins were subsequently shown to prevent AF recurrence in patients with lone AF after successful cardioversion [33] and in patients with CAD[34]. Both these studies were retrospective. However, it is well known that results obtained in retropsective studies may not be replicated in clinical trials [35]. Accordingly, in an open, controlled multicenter study, pravastatin did not reduce the recurrence rate of AF after electroversion[36]. Moreover, there has been an isolated case report of AF due to simvastatin[37], which further limits their role in the management of arrhythmias.

The evidence available for the beneficial role of statins is largely from observational and experimental studies which is clearly insufficient to recommend them as primary or even adjunctive antiarrhythmic agents. Moreover, their role in prevention as well as treatment of arrhythmias remains to be clearly defined.

### Heart failure

Initial experimental evidence indicated towards both potential harm and benefit of statins in heart failure. Statins modulate a variety of inflammatory and immune responses [38-40]. In animal models of heart failure, statins moderate abnormal collagen and β-myosin expression, attenuate increased matrix metalloproteinase activity, improve ventricular remodelling and systolic function, normalize sympathetic responses and improve survival [41-43] Given the relation of systemic inflammation to morbidity and mortality in heart failure patients, it was hypothesised that statins may benefit patients with heart failure separately from or in addition to effects on cholesterol and coronary disease[44].

In a report of 551 patients with systolic heart failure, statin use was associated with improved survival in patients with ischemic and non-ischemic heart failure[45]. After risk adjustment for age, gender, CAD, cholesterol, diabetes, medication, hemoglobin, creatinine and NYHA functional class, statin therapy remained an independent predictor of improved survival. Furthermore, in a randomised trial in 63 patients with heart failure, statin use improved NYHA class and ejection fraction when compared with placebo [46]. Also, statin therapy reduced new onset heart failure in the 4S Study [47], but this may have been related to effects on recurrent myocardial infarction. Using data from the Prospective Randomised Amlodipine Survival Evaluation (PRAISE) trial, association of statin therapy with total mortality among 1,153 patients with severe heart failure was evaluated [48]. Statin therapy was associated with a 62% lower risk of death. However, only 12% patients were receiving statin therapy. Moreover, the study results cannot be generalised as these patients participated in a clinical trial at a time when β blockers and spironolactone were not commonly used in severe heart failure.

There also is some evidence to the contrary; lower serum cholesterol predicts worse outcomes in heart failure [49], raising concerns regarding use of lipid lowering agents. Statins also reduce ubiquinone (enzyme Q-10) [50], which may adversely affect mitochondrial and cardiac muscle function.

Therefore, in lieu of conflicting experimental and clinical data, the routine use of statins in congestive heart failure will be premature.

### Cardiomyopathy (CMP)

In initial experimental studies, simvastatin was shown to induce regression of cardiac hypertrophy and fibrosis and improve cardiac function in a transgenic rabbit model of human hypertrophic CMP [51]. Based on the knowledge that statins improve endothelial function [39] and suppress systemic inflammation [31], it was hypothesized that statins may improve cardiac function in patients with nonischemic dilated CMP [46]. Fourteen weeks of treatment with simvastatin was shown to improve left ventricular ejection fraction, reduce plasma concentration of tumour necrosis factor-alpha, and brain natriuretic factor in patients with idiopathic dilated CMP[52]. The effect on patient outcomes was however not evaluated.

Again as in case of heart failure, although some evidence is available for the beneficial effect of statins in CMP, evidence to the contrary is also available. Lovastatin has been shown to significantly increase mortality in hamsters with cardiomyopathic heart due to reduction in ubiquinone supply[53]. Statins have been shown to decrease coenzyme Q levels in humans [54] and this coenzyme is indispensable for cardiac functions [55]. In wake of such conflicting evidence, their use in ishemic/nonishemic CMP cannot be advocated.

### Diabetic dyslipidemia

In addition to microvascular complications, patients with type 2 diabetes are at an increased risk of developing CAD [56] Over a 7-year period, in patients with no history of CAD, the incidence of first MI was over five times greater for patients with diabetes compared with non-diabetic controls [57]. Diabetes is now considered to be a cardiovascular disease and all diabetics, irrespective of history of CAD, are considered within the category of secondary CAD prevention. Diabetic dyslipidemia may exist in the absence of raised total serum cholesterol due to an increased proportion of the more atherogenic LDL particles. Moreover, dyslipidemia often exists with a number of other atherogenic co-factors in patients with diabetes (e.g. abdominal obesity and hyperinsulinemia) as a part of metabolic syndrome [58]. The updated Adult Treatment Panel (ATP)-III guidelines have advocated the use of statins for diabetes with or without CAD [12]. LDL lowering treatment when LDL-C is >100 mg/dL in diabetices without CAD and >70 mg/dL in diabetics with CAD has been recommended.

Since the appearance of the first report on the efficacy of statins in lowering lipid concentrations in patients with type 2 diabetes [59], clinical trial evidence has accumulated in their support as the primary lipid-lowering drugs for these patients. Subgroup analyses [60] of diabetic patients in the Antihypertensive and Lipid Lowering Treatment to Prevent Heart Attack Trial (ALLHAT-LLT) [9], the MRC/BHF Heart Protection Study (HPS) [15], and the Anglo-Scandinavian Outcomes Trial-Lipid Lowering Arm (ASCOT-LLA) [10] showed variable results of lipid lowering therapy on cardiovascular outcomes in diabetic patients. In ALLHAT-LLT [9] pravastatin did not reduce the incidence of non-fatal MI and CAD deaths in patients with diabetes. In the HPS trial [15] simvastatin significantly reduced the risk of CAD and total cardiovascular events in patients with diabetes, whether they already had CAD or not. In the ASCOT-LLA trial [10] atorvastatin did not reduce the risk of non-fatal MI and CAD death in patients with diabetes and hypertension who had no pre-existing CAD. Collaborative Atorvastatin Diabetes Study (CARDS) was carried out to evaluate the efficacy and safety of low-dose atorvastatin treatment in primary prevention of CAD in patients with type 2 diabetes at high-risk of CAD [61]. CARDS Investigators conclude that statins should be used in all patients with type 2 diabetes unless the patient has sufficiently low risk of coronary heart disease.

However, generalization of CARDS results is debatable. For example, the risk of statin therapy might be **increased **in people older than 75 years of age in patients with chronic renal insufficiency or organ transplantation and in patients with very high triglyceride concentrations who are on fibrates [60]. Moreover, the number needed to treat will be very high in patients in whom the baseline risk is low like those with type 2 diabetes who are younger than 40 years; in premenopausal women; and in those without any CAD risk factors [60].

### Development of diabetes

Lipid lowering therapy with bezafibrate had earlier shown to improve plasma glucose levels and insulin response to 75 g oral glucose loading associated with hyperinsulinema [62]. An analysis of patients enrolled in the WOSCOPS study had shown a 30% reduction in the hazard of becoming diabetic [63]. The analysis was done post hoc and the levels of statistical significance was modest (p = 0.04). Additionally, by reducing the risk of CAD, the need for β-blocker use (and perhaps thiazides) was reduced. There is some evidence that β-blockers [64,65] and thiazides [66] may be associated with an increase in the incidence of diabetes.

Although no effect of pravastatin on glucose levels was shown in another study, [67] the authors proposed that pravastatin might reduce the incidence of diabetes by a reduction in triglyceride (TG) levels. However, even this is unlikely because the effect of pravastatin on TG levels is only modest [68]. A recent case control study from the UK based General Practice Research Database failed to show reduced incidence of development of diabetes [69].

### Diabetic maculopathy

There has been interest in link between serum lipids and retinal exudates for 40 years [70]. A number of cross-sectional studies suggest that serum lipids may have a causative role in the formation of macular exudates [71-74]. A cross-sectional study of Age-related Macular Degeneration (AMD) suggests that statin therapy does have a protective role against the development of macular degeneration [75].

Few studies have evaluated statins in diabetic retinopathy [76,77]. In one of these, an improvement in hard exudates was noted in all patients on statins [76]. In another study, simvastatin was shown to improve fluoroscein angiographic picture and led to maintenance of visual acuity in all patients [78].

These data, though important, do not permit us to draw a final conclusion as these studies were inadequately powered.

### Claudication

Claudication occurs when blood flow to the extremity fails to meet the metabolic demands of the skeletal muscle during exercise. It was hypothesised that statins, by improving endothelium dependent vasodilation at the arteriolar and capillary level [79], by their proangiogenic response independent of cholesterol reduction [80], and by inhibition of MMP-9 secretion by peripheral monocytes [81], could be beneficial in reducing claudication in patients with peripheral arterial occlusive disease (PAOD). Studies with lipid modifying therapies have demonstrated desirable effects in patients with PAOD [82,83]. A post-hoc analysis of the 4S data showed that new or worsening claudication was reduced in the group of patients receiving statins [84]. High-dose, short-term therapy with simvastatin has been shown to improve walking performance, ankle-brachial pressure indices, and symptoms of claudication in hypercholesterolemic patients with PAOD [85]. One-year treatment with atorvastatin improved pain free walking time and participation in physical activity in patients with intermittent claudication [86]. However, maximal walking time did not change significantly. Similar benefit was shown with simvastatin on treadmill exercise time until the onset of intermittent claudication [87].

Despite the evidence from these studies suggesting benefit, well-designed long-term studies assessing primary and secondary prevention of PAOD with defined endpoints such as amputation or number of vascular events are required.

### Multiple sclerosis (MS)

In an experimental model of encephalomyelitis, lovastatin treatment was shown to block disease progression and induction of inflammatory cytokines [88]. Lovastatin treatment also attenuated the transmigration of mononuclear cells by downregulating the expression of leukocyte function antigen-**1 **(LFA-1), a ligand for intercellular adhesion molecule (ICAM), in endothelial-leukocyte interaction [88] and mononuclear cell infiltration into the CNS has been implicated in MS [89]. Atorvastatin was shown to promote Th2 bias and reverse paralysis in a CD4(+)Th1-mediated experimental model of MS [90].

Therefore, statins were recognised as potential agents for future pharmacotherapy of MS [91]. In the first clinical trial of statins in MS, 80 mg oral simvastatin for 6 months significantly reduced the number and volume of gadolinium enhancing lesions [92]. However, immunological expression of surface markers on leukocyte cells or inflammatory cytokine profile showed no changes. Moreover, it was an uncontrolled, open label, small study with a baseline versus treatment comparison. Therefore, its results must be interpreted with caution. For instance, it is possible that reduction in the disease severity as measured with MRI could be due to regression to the mean. Moreover, since patients were included on the basis of the presence of gadolinium enhancement, this might have led to selection of patients with active disease who may subsequently have shown spontaneous reduction in disease activity anyhow. Additional factors like steroid use and unblinded assessment of MRI scans may have influenced the results. The exploratory immunological data in this study were also not found to be supportive.

Due to the paucity of evidence from adequately powered good quality clinical trials demonstrating the benefits of statins, any conclusive statement would be rather premature. Several trials are currently underway to address this question and we are also conducting a Double-blind, Randomised Evaluation of Atorvastatin in Multiple Sclerosis (DREAMS) trial in our institution.

### Stroke

Although cholesterol lowering is well known to decrease the risk of CAD, its association with decreased risk of stroke was demonstrated later [93]. Meta-analyses done recently have shown statin use to be associated with reduced risk of stroke by 12 to 24% [94,95].

Analysis of data from nine cohort studies showed a 15% decrease in thromboembolic stroke but a 19% increase in hemorrhagic stroke for a 1.0 mmol/l decrease in LDL concentration. The risk in those without a known cardiovascular risk factor was shown to be the same (6%) in clinical trials as that seen in cohort studies [91]. Though the overall risk of non-fatal strokes was reduced, the risk of fatal strokes was not [96]. Also, these results were obtained from studies which had stroke as their secondary endpoint. Moreover, in most of the included studies, incidence of stroke was very low, especially for primary prevention, reducing the power of comparison.

### Alzheimer's disease (AD)

Addition of lovastatin to human HEK cells transfected with the amyloid precursor protein (APP) was shown to reduce intracellular cholesterol/protein ratios by 50%, and to inhibit cleavage of APP by beta-secretase [97]. Non-demented individuals with heart disease have increased prevalence of AD-like beta-amyloid deposits in the neuropil and within neurons [98]. In a cohort of patients taking lovastatin and pravastatin (but not simvastatin), a lower prevalence of diagnosed probable AD was noted [99]. A case control study has also shown a lower risk of dementia among users of statins [100].

However, in a review done by the Cochrane Group, it was pointed out that no evidence in the form of controlled clinical trials was available to recommend the use of statins in AD [101]. In a subsequent randomised, placebo controlled, double-blind trial, 26-week treatment with 80 mg simvastatin did not show any significant alteration in the cerebrospinal fluid levels of A-beta 40 and A-beta 42 [102]. Though the body of evidence for the beneficial effect of statins for AD is growing, due to the paucity of randomised controlled trials, no conclusions can yet be drawn [103].

Moreover, excessive lipid lowering may be detrimental as too little cholesterol in neural membranes has been shown to increase the vulnerability of neural membranes to dysfunction [104]. Low serum cholesterol concentrations have been shown to be associated with cognitive decline in prospective studies of aging American twins [105] and elderly Finns [106].

### Depression

Two observational studies showed that long-term statin use is associated with a reduced risk of depression in patients with CAD [107,108]. After an average follow up of 4 years, comparison of psychometric scores between users and nonusers of statins showed that statin use was associated with lower risk of abnormal scores for depression, anxiety and hostility [107]. Authors have attributed the findings to a possible direct effect of statins on psychological well being. Similar reduced risk of depression was noted with statins in patients with hyperlipidemia [108]. A more plausible possibility of reduced risk of depression due to an improvement in the overall quality of life was suggested in this study.

On the other hand, lowering of serum cholesterol may be associated with an increased incidence of depression and suicides [109-113]. To sort of neutralize the evidence, some randomised, placebo controlled trials of statins have shown that depression was neither more nor less common among those taking active treatment [114-116].

### Rheumatoid arthritis (RA)

Statins were shown to inhibit LFA-1, which is known to play an important role in the pathophysiology of inflammatory and autoimmune diseases [117]. Statins also led to significant suppression of collagen-specific Th 1 humoral and cellular immune responses, reduction of anti-CD3/anti-CD28 proliferation and IFN-gamma release from mononuclear cells derived from peripheral blood and synovial fluid [118]. Based on these findings, a putative role for statins in RA was suggested.

A preliminary study done in 15 patients with RA who were receiving methotrexate as a single disease modifying agent with no satisfactory responses, showed improvement after eight weeks of treatment with 40 mg simvastatin [119]. Recently, in a randomized placebo controlled trial [120], atorvastatin 40 mg was shown to significantly improve disease activity score after 6 months of therapy although the effects were modest. The use of disease modifying anti-rheumatic drugs was rather heterogeneous among the treatment groups in this study, with more patients receiving methotrexate in the atorvastatin group. Other limitations were a small study group and a direct effect of statins on hepatic CRP synthesis, which could exaggerate the impression of disease modification.

### Osteoporosis

The biologic effects of statins on bone metabolism have been reported in literature [121]. Statins were shown to be potent stimulators of bone formation *in vitro*. Statins were shown to stimulate the bone morphogenic protein-2 (BMP-2) promoter in an immortalized osteoblast cell line [121]. BMP-2 is known to enhance osteoblast differentiation [122]. Further supporting evidence for its beneficial role came from osteoporosis observational studies [123-126]. However, in these studies, no adjustment for weight was made and part of the protective effect of statins could be because of reduction in weight.

By contrast, the Women's Health Initiative Observation Study found no relationship between statins and hip/wrist/arm/non-spine fracture rates after adjusting for weight and other potential confounders [127]. Lack of benefit of statins in reducing hip and non-spine fracture was also reported in a case control study from the General Practice Research Database [128]. In the first placebo-controlled trial specifically designed to assess bone turnover, statin treatment did not show any difference in rates of bone formation [129]. Other uncontrolled studies have been conflicting; both increased [130] and decreased [131,132] rates of bone formation have been reported. In spate of high optimism, it has been suggested that increasing the bioavailability of statins to the bone may lead to better results [133]. As of now, keeping in mind lack of a consistent response with statins in various studies, it will be inappropriate to conclude that statins have a meaningful benefit for patients with osteoporosis.

### Cancer

Similar to most of the above mentioned indications, the action of statins in cancer has been bi-fold with arguments and evidence both in favour and against having been published.

It was suggested, nearly a decade ago, that cholesterol inhibition could inhibit tumour cell growth and possibly prevent carcinogenesis [134]. Recently, statin use was shown to be associated with a reduced risk of breast [135] and colorectal [136] carcinoma. However, these findings need confirmation as they were based on a small number of events. Statin use has been associated with a 20% reduction in colon cancer, if used for more than 4 years and if more than 1350 defined daily doses were taken [136].

Evidence to the contrary has also grown simultaneously. Epidemiological studies in the early 1990s had shown a rise in non-cardiovascular mortality, particularly cancer deaths in people with low cholesterol concentrations [137]. Similar conclusions have been drawn from results of early trials of cholesterol lowering [138]. Some researchers have shown that lipid-lowering drugs, including statins, increase the occurrence of several types of cancer in rodents [139]. In the CARE trial [6], incidence of female breast cancer and in the PROSPER trial [8] in elderly, incidence of all cancers increased in patients given pravastatin.

With such conflicting evidence available there is a need for exercising cautious scepticism for a potential beneficial role of statins for cancers.

### Acquired Immune Deficiency Syndrome

Hyperlipidemia induced by antiretroviral treatment is observed frequently and can cause an increase in cardiovascular risk in HIV patients [139]. Moreover, HIV infection itself induces pro-atherogenic lipid changes, which may lead to an increased cardiovascular risk but are partly reversed by antiretroviral regimens [140]. Statins, given to patients with HIV infection and hyperlipidemia, effectively reduced total cholesterol (27%) and triglycerides (15%) [141]. In the first double-blind, placebo-controlled study of the effects of statin therapy on lipids, lipoprotein subfractions, and endothelial function in HIV patients taking protease inhibitors, pravastatin reduced concentrations of atherogenic lipoproteins [142]. Similar beneficial effects of statins were shown in a cohort of 245 patients [143]. However, in all these studies the decrease in total cholesterol, LDL and triglycerides was only modest, and a significant number of patients did not achieve their NCEP goals. Moreover, the risk of rhabdomyolysis with concomitant use of statins in patients receiving highly active anti-retroviral therapy needs to be carefully evaluated in future studies.

Statins have been shown to have a direct effect on HIV infection itself [144,145]. In *in vitro *studies, 9 days after viral loading, lovastatin inhibited both sterol synthesis and viral multiplication in Human H9 lymphocytic cell line [144]. Rho-guanosine triphosphatase (GTPase) activity is required for HIV infectivity into the cells [145]. Statins block Rho-A activation induced by HIV-1 binding to target cells and also inhibited entry of HIV-1 pseudotyped viruses. These data are only experimental and considerable work will need to be done before any speculations for anti-retroviral potentials of statins are made.

### Other indications

Some of the other uses for which statins are being evaluated are drug-induced dyslipidemia following transplantation [146,147], for causing immunosuppression in patients undergoing organ transplantation [148], promotion of fracture healing in vascularised bone allograft [149], sickle cell anemia [150,151], idiopathic pulmonary fibrosis [152,153], sensorimotor recovery after experimental intra-cerebral haemorrhage [154], sepsis [155-157], and glomerulonephritis [158]. However, only limited, preliminary data are available to support routine use of statins in most of these indications and no recommendations can be made at present.

### Safety issues

One cannot ignore the safety concerns with statin use; besides the well known side effects of myopathy, procarcinogenesis potential [159,160], nerve damage [161,162], short temper [163], cognitive decline [164], memory loss [165], teratogenic potential [166,167], and more recently loss of libido [168] are some of the other concerns.

### The rise, plateau and fall (?) of statins

There is no doubt that statins have become one of the most commonly utilized drugs in cardiac patients not only in developed [169] but also in developing nations [170]. It is also obvious that their use will be intensively promoted in many non-cardiac conditions discussed above although the tremendous promise seen in some experimental and initial clinical studies failed to be sustained in clinical trials or if it did the effect was only modest. For others the initial conflicting results continue to exist.

Recent years have shown a kind of contagiousness being demonstrated in research. Foremost among these have been the case of COX-2 inhibitors. After the discovery of COX-2 isoenzyme, almost every pathophysiological process showed involvement of COX-2 [171,172]. Selective inhibition of COX-2 was thought to be the answer to a number of problems in therapeutics. A large number of studies giving evidence to the contrary or addressing adverse effects of COX-2 inhibitors got overshadowed (or were suppressed) in the hype created over COX-2 inhibitors [173,174]. Rofecoxib and some other selective COX-2 inhibitors are being withdrawn for their adverse effect profiles as their discoverer companies gear up for payments of compensation claims made by sufferers. Many other molecules have suffered similar fate and we hypothesized that statin research may also be on decline.

To test this hypothesis we searched Medline using the MeSH term "statins", "statins AND cancer (as well as other indications one by one)" for overall and yearwise extraction of citations. A total of approximately 11,000 citations were found out of which about 50% have appeared in only the past 4 years (since our last review [2]). An analysis of yearly trends showed some interesting details. The first study on statins was reported in 1975 [175]. Subsequently, there was a steady increase in the publications until pleiotropism of statins was suggested in the mid-90s [176] and since then (especially since 2000), a steep rise in publications for various indications with a peak around 2002–2003 can be noticed. It is interesting to note that a trend towards a decline in the number of these studies can already be seen for statins in general (Fig 1) and in many indications specifically (Fig 2). This declining trend is probably due to failure to establish any definite benefit in majority of the indications for which their use was proposed.

**Figure 1:**
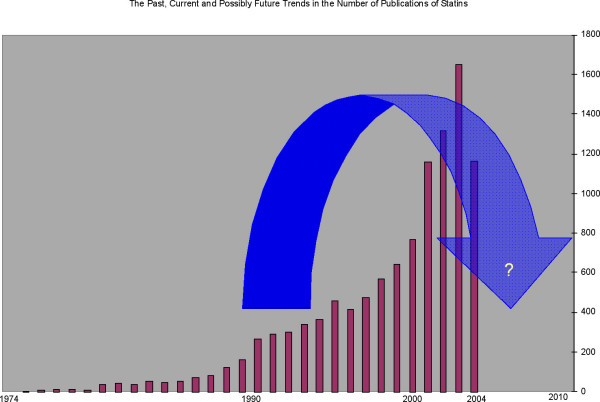
Number of statin publications in each year from 1974 to 2004. The numbers depict the citations obtained from Pubmed on entering the MeSH term 'Statins'

**Figure 2:**
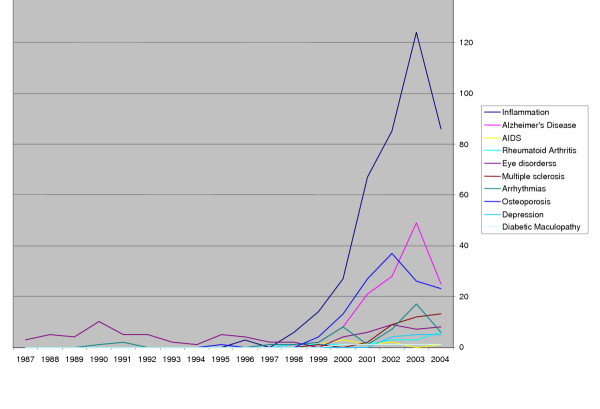
The trend in the number of published research articles in Pubmed, categorized according to the various pathological conditions discussed in the text.

Therefore, our hypothesis which appeared quite implausible initially may not have been altogether wide of the mark. Consequently, it remains to be seen whether statins can withstand the test of time or will sink into oblivion like many of the other molecules.

## Conclusion

If we take an overview of the evidence available for each of the above indications of statins we notice that it is rather weak even for the indications in which there are controlled trials available. Moreover, these trials are either inadequately powered or have measured only soft endpoints or have been of short duration to be conclusive. And lastly, a considerable number of contradictory studies make their utility in most of these diverse conditions doubtful.
